# Pets, Wildlife and Parasites

**DOI:** 10.3390/pathogens12111310

**Published:** 2023-11-02

**Authors:** Anastasia Diakou, Georgiana Deak, Fabrizia Veronesi

**Affiliations:** 1 Laboratory of Parasitology and Parasitic Diseases, School of Veterinary Medicine, Faculty of Health Sciences, Aristotle University of Thessaloniki, 54124 Thessaloniki, Greece; 2 Department of Parasitology and Parasitic Diseases, Faculty of Veterinary Medicine, University of Agricultural Sciences and Veterinary Medicine of Cluj-Napoca, 400372 Cluj-Napoca, Romania; georgiana.deak@usamvcluj.ro; 3 Parasitology Laboratory of the University Teaching Hospital, Department of Veterinary Medicine, University of Perugia, 06124 Perugia, Italy; fabrizia.veronesi@unipg.it

## 1. Introduction

In our dynamic world, borders of different sorts are being rapidly altered or even erased. Environmental, climatic, socioeconomic, geopolitical and behavioural changes are at the core of this transformation. The spread or sharing of pathogens between different areas of the world, different environments and different animal species is a characteristic manifestation of this phenomenon, with the COVID-19 pandemic holding a prominent place as an example.

Parasites are among the most prevalent health-impairing agents, affecting both pet animals and wildlife. Even though parasitism is the norm in wild animals, it is considered an unacceptable symbiosis in pet animals. Therefore prevention and treatment are implemented to minimize parasitic infections and infestations. Regardless of the affected animal species and domestication status, parasites may severely affect animals’ health, and parasitic diseases are occasionally fatal. Additionally, zoonotic parasitic diseases are a known threat to human health, and human infection can occur independently of a given individual’s contact with pets or wild animals via food, water, soil, or vectors.

Pet ownership is presently a common and popular practice in most parts of the world. It has been estimated that 50% of people in all developed countries keep at least one pet, with dogs and cats being the most popular choice among pet owners. As can be expected, for pet owners, the health and wellbeing of the animals is a primary concern; however, widespread ownership also triggers discussion concerning zoonotic diseases.

On the other hand, wild animals have a particular—and occasionally decisive—role in maintaining and spreading infectious agents; knowledge of this role is essential in research that focuses both upon their health/conservation status and on their contribution to the epizootiology of important pathogens, which may spread to domestic animals and/or to humans.

In this stimulating context of dynamic changes and interactions related to the transmission and spread of pathogens, the journal “Pathogens” has launched a Special Issue entitled “Pets, Wildlife and Parasites”. The primary objective of this Special Issue was to present novel insights and information to the scientific community through the publication of articles that focus on parasites and parasitic diseases affecting both pets and wild animals. The articles in this Special Issue address topics such as the epizootiology of these diseases, including aspects like occurrence, prevalence, distribution, and new host records. Furthermore, this Special Issue delves into the impact of parasitic diseases on the health and conservation status of wild animals, and it explores the presence of evidenced or potential bridging infections, which could have implications for the health of multiple species. Another important aspect addressed is the zoonotic potential of these diseases, shedding light on possible transmission to humans with regard to the concept of One Health and recognizing the interconnectedness of animal, human, and environmental health.

This editorial summarizes the information provided in this Special Issue, through the publication of ten articles, i.e., six research papers, three case reports and one review. These articles provide valuable insights regarding different parasitic infections in various animal species, expanding our knowledge regarding epizootiology, geographical distribution, pathogenesis, and the impact of parasites in a changing world.

## 2. Pets

Three of the papers published in this Special were surveys investigating parasitic infections of dogs.

The case reported by Blaga et al. [1] describes the first incidence of cutaneous lesions caused by concomitant *Toxoplasma gondii* and *Alternaria* spp. infection in a dog receiving immunosuppressive treatment. *Toxoplasma gondii* is one of the most common parasites among animals and humans [2]. The infection is commonly asymptomatic but clinical signs, which occasionally can be severe, may develop in the acute phase or during the reactivation of the disease, a condition usually observed in immunosuppressed or immunocompromised individuals [2]. Although dogs are frequently *T. gondii*-seropositive, there have only been a few clinical cases reported in these animals, mostly concerning pulmonary and neurological implications [2].

Cutaneous lesions in dogs due to toxoplasmosis are quite rare [3]. In the case discussed here [1], 1 cm in diameter, non-pruritic, suppurative nodules and round alopecic nodules were observed in a dog under immunosuppressive therapy. The diagnostic approach included fine-needle aspiration cytology, histology, serology and PCR-coupled sequencing, confirming the presence of *T. gondii* and *Alternaria* spp. In this paper, the authors place special emphasis on discussing diagnostic options, considering that the clinical signs of cutaneous toxoplasmosis are not specific. Various diagnostic methods can indicate *T. gondii* infection; however, the parasite’s presence as the causative agent of cutaneous lesions requires confirmation via PCR, ensuring differential diagnosis from other infections with a similar cytological and histological appearance, e.g., neosporosis.

The clinical condition was resolved through the administration of clindamycin and a reduction in the immunosuppressive drug dosage. The co-infection with *T. gondii* and *Alternaria* spp. draws attention to the possibility of opportunistic concomitant infections in immunosuppressed animals. Considering this, the authors suggest that before starting immunosuppressive therapy, it is advisable to conduct serologic testing for toxoplasmosis or neosporosis in dogs and cats. Additionally, it is suggested that these animals have regular dermatological assessments throughout the duration of their immunosuppressive therapy.

*Dirofilaria immitis* is a mosquito-transmitted nematode, causing heartworm disease. Dogs are the primary definitive host of this parasite; however, other mammals can also be infected. Consequently, infected dogs can introduce the parasite to previously free areas and, more importantly, to endangered populations of wild animals, establishing an critical conservation threat. The article by Culda et al. [4] explores such a case, by investigating the presence of *D. immitis* in dogs on San Cristobal Island, Galapagos, the homeland of the endemic and endangered Galapagos sea lion (*Zalophus wollebaeki*). Considering that *D. immitis* has been reported in other pinnipeds, causing occasionally fatal disease [5], the presence of microfilaraemic dogs in the area could represent a source of infection for Galapagos sea lions and thus a threat to their endangered population. According to the results of the study [4], 1.7% of the examined dogs bore *D. immitis* microfilariae in their blood circulation. The authors propose a series of steps for further research on the threat of heartworm disease in Galapagos sea lions; these include extended epizootiological investigations in all Galapagos islands, and studies on heartworm transmission, i.e., the mosquito vector species involved, their preferred source of blood meal and the possible role of Galapagos sea lions as reservoirs for *D. immitis*.

Monitoring the parasitofauna of dogs in an area is of interest, not only for the sake of dogs’ health protection, but also for the surveillance of health threats to other animals and humans. In their study, Safarov et al. [6] aimed to determine the diversity and prevalence of helminths in dogs from the Tashkent, Samarkand and Karakalpakstan regions of Uzbekistan. Dogs from both rural and urban areas were included in the study, reaching a total of 399 animals. A strength of this paper is the examination of the animals via necropsy, providing the advantage of helminth morphological identification, which can be more accurate compared to identification via the parasitic elements found in faecal examination. The results revealed the presence of 31 helminth species in 94.7% of the examined dogs. It should be highlighted that 18 of those parasites are zoonotic. Among them, *Echinicoccus granulosus*, *Dipylidium caninum*, *Toxocara canis* and *Dirofilaria repens* are of particular importance as they are commonly found to affect humans, occasionally causing severe disease. As expected, a comparison between the areas revealed that dogs in rural areas are more frequently infected and bear a higher diversity of helminths than urban dogs. The results clearly indicate the need for raising public awareness regarding the importance of regular veterinary care including antiparasitic treatments, both in owned and in stray dogs.

## 3. Wildlife

Six papers on various parasites found in wild animals were published in this Special Issue.

*Thelazia callipaeda*, the “oriental eye worm”, is a vector-transmitted nematode parasite that is distributed across Europe and Asia. Domestic and wild carnivores are the main hosts, but *T. callipaeda* has also been identified in lagomorphs, wild boars, and humans [7]. In Romania, thelaziosis was diagnosed for the first time in 2015, in a domestic dog [8]. Since then, it has been reported in other hosts, namely cats, wild canids (jackals, wolves, foxes), wildcats, mustelids, and lagomorphs (rev. in [9]). Considering that the latter are abundant in Romania, Cotuțiu et al. [9] investigated the presence of *T. callipaeda* in wild hares from Romania, in order to determine whether these animals could represent a potential reservoir host in the country. According to the results, the prevalence of *T. callipaeda* in Romanian hares is relatively low (1.23%), while a genetic analysis confirmed the presence of haplotype 1, the only haplotype identified in Europe to date. This is the first report of *T. callipaeda* in European brown hares in Romania and the second report in Europe, emphasizing the potential role of this animal species as a reservoir host.

In addition to the oriental eye worm, European brown hares can harbour a wide diversity of endoparasites, including protozoans, trematodes, cestodes and nematodes. The survey performed by Brustenga et al. [10] in Italy on 215 faecal samples from European brown hares bred for restocking purposes showed that the most prevalent parasites belonged to the genus *Eimeria* (91.2%), followed by the nematodes *Trichostrongylus retortaeformis* (21.4%), *Passalurus ambiguous* (9.3%), and *Strongyloides papillousus* (6.5%), the cestode *Cittotenia* spp. (5.6%), and the trematode *Dicrocoelium dendriticum* (1.4%). The authors suggest that similar studies in the congeneric endemic Italian hare (*Lepus corsicanus*) and Sardinian hare (*Lepus capensis mediterraneus*) would be useful for health control and the successful breeding of these species, in an effort to conserve these vulnerable endemic animal species.

An interesting case of ovarian filariosis in a road-killed, adult southern tamanduas (*Tamandua tetradactyla*) is described in the report by Fromme et al. [11]. The parasites could not be molecularly identified, but their morphological characteristics were consistent with nematodes of the superfamily Filarioidea. The infection was associated with severe lesions of the ovaries, which were enlarged, with multiple nodules enclosing the nematode parasites. Parasitosis of the ovaries is a rare condition in all kinds of mammals [12]; however, in southern tamanduas, ovarian filarial nematodes have been also described in the past [13], which makes this recent report [11] particularly interesting andgives prominence to ovarian filariosis as a possible reproductive hindrance in this animal species. Further studies on this parasitosis in southern tamanduas and other Xenarthra are recommended, especially in the context of conservation projects focusing on the vulnerable or near-threatened species among them.

*Taenia crassiceps* is a widely distributed parasite that predominantly circulates between canines and rodents, which act as definitive and intermediate hosts, respectively. Rarely, dogs may also act as intermediate hosts, developing subcutaneous cysticercosis as the most common clinical presentation. *Taenia crassiceps* is a parasite of zoonotic significance, as humans may act as accidental intermediate hosts [14,15,16]. The article by Zhang et al. [17] documents the occurrence of cysticercosis caused by *T. crassiceps* in a muskrat (*Ondatra zibethicus*) and in a domestic dog from the northeastern United States (New Jersey). The infection was identified via parasitological and histopathological examinations in both cases, while in the case of the muskrat, a molecular identification of the parasite species was also performed. Cysticercosis caused by *T. crassiceps* in dogs had already been described in the same area, but it was considered a rare disease; the first description of muskrat infection in the area, by Zhang et al. [17], suggests that veterinarians and medical doctors should increase their awareness regarding this parasite in order to achieve timely diagnosis, interventions and surveillance, in relation to the “One Health” concept.

Dogs are the natural host and reservoir of canine heartworm, i.e., *Dirofilaria immitis.* However, this parasite can also infect over 30 mammalian species, although patent infections in these hosts have rarely been documented [5,18,19]. Romania is an enzootic country for canine heartworm [20,21,22], and *Dirofilaria* spp. infections had been detected in the past via necropsy and/or molecular tools in six species of wild carnivores throughout the country [23,24]. The article by Ionica et al. [25] describes the results of a survey on the presence of *D. immitis* carried out in Romania, via necropsy, in 459 wild carnivores belonging to 17 different species. The results of the study revealed an overall prevalence of 4.36%. The infected animals were twelve golden jackals (19.05%), four red foxes (6.67%), one raccoon dog, two wild cats (4.65%) and one European badger (0.87%). Interestingly, only one golden jackal and the European badger were microfilaraemic. The study provides further evidence of the occurrence of *D. immitis* in Romania, expanding the known host spectrum. Crucially, this survey reports a new host species for this parasite, the European badger, and a new host for Europe, the raccoon dog.

*Toxoplasma gondii* and *Neospora caninum* are two protozoan parasites of major significance for livestock, while *T. gondii* is also a common and major threat to public health [26]. Both parasites have a wide range of intermediate hosts, including birds [27]. In particular, birds of prey are highly exposed to the horizontal transmission of both parasites, by feeding on infected small mammals and other birds, or through the consumption of sporulated oocysts in contaminated water or food sources. Thus, they play a critical role in the maintenance and spread of these parasites in nature. The article by Zanet et al. [27] investigates the prevalence of *T. gondii* and *N. caninum* in 159 migratory and non-migratory birds of prey, belonging to 19 species of the Strigidae, Accipitridae, Tytonidae and Falconidae families, recovered across Central Italy. The skeletal muscle and myocardium of the necropsied animals were molecularly examined and *T. gondii* genotyping via multi-locus PCR-RFLP was performed. An overall prevalence of 5.66% for *N. caninum* and 23.27% for *T. gondii* was recorded, with the type I strain of *Toxoplasma* being the preeminent, followed by the type II strain; furthermore, atypical strains were found in two isolates. Based on the results, the circulation of both parasites in birds of prey was confirmed in the studied area, while tawny owl (*Strix aluco)* and long-eared owl (*Asio otus)* were included for the first time in the list of potential intermediate hosts of *N. caninum*.

## 4. The Wildlife–Domestic Animals–Human Interfaces

The review article entitled “Wild mesocarnivores as reservoirs of endoparasites causing important zoonoses and emerging bridging infections across Europe” [28] calls attention to the accumulated knowledge regarding the role of wild mesocarnivore, i.e., small and mid-sized (<15 kg) wild carnivores, in the emergence and re-emergence of important parasites. Interestingly, mesocarnivores display particular traits that render their epidemiological role in parasite maintenance and transmission exceptional. Most of these animals have an omnivorous diet and exhibit a broad preference for various habitats. As a result, they frequently reside near human settlements, positioning themselves at the apex of the food chain without facing any competitive pressure from other animal species. Due to their relatively small size and adaptable nature with regard to thriving in diverse habitats, these animals are plentiful in a wide array of environments. They often develop their activity in proximity to humanised environments, a fact that has been intensified in recent years as a result of land use changes and the overall destruction and fragmentation of wild habitats.

The article is organised on an animal group or animal species basis, and discusses the most significant parasites encountered in the red fox (*Vulpes vulpes*), golden jackal (Canis aureus), common raccoon dog (*Nyctereutes procyonoides*), raccoon (*Procyon lotor*), European wildcat (*Felis silvestris*), the genera *Meles*, *Martes*, *Mustela*, and *Lutra*, and the American mink (*Neogale vison*). In this context, over 21 different parasites, listed in the Table of the article, that infect mesocarnivores are discussed. As all these parasites can be transmitted to domestic animals and as many of them are of zoonotic significance, continuing investigations and monitoring are recommended for the timely prevention and control of their spread.
